# Swimming for Women

**Published:** 1859-01

**Authors:** 


Swimming for Women.

*Why do not Women Swim? A Voice from many Waters,* is the title of one of the tracts issued by the same association.

"When shall we," says the author, "leave off our wild goose chase after health in the consulting rooms of charlatans, and know that she lies hidden in the bright wave and shady glen, and sits wooing us among the flowers on the meadow-side? In vain, for most of us, the fresh mountain breeze blows; in vain the fields are green and beautiful; in vain volumes of health-giving water circle our island home; we value them them little, because of their very freeness."

The instruction of women in swimming is advocated, on the ground that the ability to perform this exercise would be the means both of saving life in shipwreck and other accidents on the water, and of developing the full hygienic value of our seas and rivers. To the objections that "swimming is utterly inconsistent with womanly decorum," and that it "has a tendency to make the female sex masculine," answers are given. We do not like "strong-minded" women of the *ultra* stamp; but surely there is room for an advance beyond the close conventional limits of modern civilisation. Woman would not be less woman if she could swim.

